# Optimized Leaky-Wave Antenna for Hyperthermia in Biological Tissue Theoretical Model

**DOI:** 10.3390/s23218923

**Published:** 2023-11-02

**Authors:** Alessandro Calcaterra, Patrizio Simeoni, Marco Donald Migliore, Fabio Mangini, Fabrizio Frezza

**Affiliations:** 1 Department of Information Engineering, Electronics, and Telecommunications, “La Sapienza”, University of Rome, Via Eudossiana 18, 00184 Rome, Italy; 2 National Transport Authority (NTA), Harcourt Lane, D02 WT20 Dublin, Ireland; 3 Dipartimento di Ingegneria Elettrica e dell’Informazione (DIEI) “Maurizio Scarano”, University of Cassino and Southern Lazio, 95123 Cassino, Italy

**Keywords:** inhomogeneous waves, leaky waves, leaky-wave antennas, hyperthermia

## Abstract

In this paper, we exploit the enhanced penetration reachable through inhomogeneous waves to induce hyperthermia in biological tissues. We will present a leaky-wave antenna inspired by the Menzel antenna which has been shortened through opportune design and optimizations and that has been designed to optimize the penetration at the interface with the skin, allowing penetration in the skin layer at a constant temperature, and enhanced penetration in the overall structure considered. Past papers both numerically and analytically demonstrated the possibility of reducing the attenuation that the electromagnetic waves are subject to when travelling inside a lossy medium by using inhomogeneous waves. In those papers, a structure (the leaky-wave antenna) is shown to allow the effect, but such a radiator suffers from low efficiency. Also, at the frequencies that are most used for hyperthermia application, a classical leaky-wave antenna would be too long; here is where the idea of the shortened leaky-wave arises. To numerically analyze the penetration in biological tissues, this paper considers a numerical prototype of a sample of flesh, composed of superficial skin layers, followed by fat and an undefined layer of muscles.

## 1. Introduction

Microwave hyperthermia is a widely utilized technique in cancer treatment [1]. In simple terms, hyperthermia involves delivering a precise amount of energy to the targeted tissue, resulting in a controlled temperature increase. Microwaves offer a unique advantage over other methods by enabling heating in the volume of tissue, leading to a more even temperature distribution. The temperature profile is a crucial factor in hyperthermia, as non-microwave approaches relying on thermal diffusion tend to overheat the skin surface, whereas microwaves distribute energy within the tissue, resulting in lower skin surface temperatures. The release of microwave energy diminishes exponentially with tissue depth, making it essential to achieve a “low exponential decay” of absorbed power to enable deeper microwave penetration. This paper aims to describe a novel type of microwave applicator that facilitates deeper penetration by generating an appropriate inhomogeneous plane wave within the tissue that optimizes the penetration angle with the skin tissue. The applicator is based on a leaky-wave structure, which has been profoundly modified to meet two additional important requirements of microwave applicators: high efficiency (i.e., effectively delivering a substantial portion of the input power to the tissue) and treatment coverage of a significant tissue area. To fulfill these objectives, the original leaky-wave structure is bent, forming two parallel structures that are fed via a Wilkinson power divider. Through analysis with commercial full-wave simulators, the final structure is proven to be compact, efficient, and capable of effectively releasing energy within the tissues.

The introduction section of this article is followed by a subsection dedicated to the theoretical background, provided to allow the understanding of the theory behind this antenna design. In Section 2, the design is explained and the chosen numerical models for the biological tissues are given. Finally, the simulation environment and the positioning of the model relative to the antenna have been described. In Section 3, the results of the simulation are illustrated, and in Section 4, the importance of the design proposed is highlighted, but also the compromises made are given, and objectives for future research are indicated. Finally, conclusions are provided.

### Theoretical Background

It has been demonstrated in the literature that the incidence of an inhomogeneous wave incoming from a non-dissipative medium on a lossy medium may produce a transmitted wave that can penetrate deeper than the one produced by the incidence of a more conventional homogeneous wave, both numerically [2] and analytically [3,4,5,6]. In order to obtain a deep-penetration effect, the incoming wave must fulfill some conditions that bind the minimum attenuation vector to the electromagnetic characteristics of the medium and the incidence angle [5]. In fact, the minimum attenuation vector for which it is possible to achieve deep penetration is

(1) $$\alpha_{c}=\frac{k_{1}}{\sqrt{2}}\sqrt{-1+\sqrt{1+{\left[\frac{2ℑ\left(k_{2}^{2}\right)}{k_{1}^{2}}\right]}^{2}}}$$

where $k_{1}=\sqrt{{\underline{k}}_{1}\cdot {\underline{k}}_{1}}$ and $k_{2}=\sqrt{{\underline{k}}_{2}\cdot {\underline{k}}_{2}}$ are, respectively, the wave vectors in the lossless medium and in the lossy medium. In correspondence of those values of the attenuation vector, it is either [5]:

(2) $$\xi_{c}=\frac{1}{2}\arcsin\left[\frac{ℑ\left(k_{2}^{2}\right)}{\beta_{1}\alpha_{1}}\right]$$

or

(3) $$\xi_{c}=\frac{\pi }{2}-\frac{1}{2}\arcsin\left[\frac{ℑ\left(k_{2}^{2}\right)}{\beta_{1}\alpha_{1}}\right]$$

depending on the characteristics of the media involved, where ξ is the angle formed by the phase vector with the normal to the interface between the air and the lossy medium and the subscript “c” stands for “critical”, meaning that $\xi_{c}$ is the minimum value of ξ that assures the deep-penetration phenomenon, i.e., $\zeta_{2}=90^{\circ }$, $\zeta_{2}$ being the angle formed by the attenuation vector of the wave inside the lossy medium with the normal to the interface with the air [5]. Moreover, in [6], an alternative and equivalent description, more suitable for ray tracing techniques, has been developed, and, among the results, it is demonstrated that the deep-penetration effect can be achieved at the interface between two lossy media. The deep-penetration phenomenon requires an incidence angle different from $0^{\circ }$, and in any case, the presence of an attenuation vector in the incidence wave constitutes a sufficient condition for a possible enhancement of the penetration in a lossy medium, i.e., even in case of normal incidence, an inhomogeneous wave may allow deeper penetration [2]. It has to be noted, by the way, that a less attenuated field does not necessarily correspond to a stronger field inside the medium, or at its surface. In practical applications, such as microwave hyperthermia, the absolute value of the field within the lossy medium is of main interest. On the other hand, a great attenuation means that the field strongly loses power with respect to a less attenuated field, so, to reach the desired field at a certain depth, a stronger field at the surface (i.e., the skin) may need to be imposed, thus resulting into overheating or burning of the surface. The inhomogeneous wave behavior promises a more homogeneous distribution of the field amplitude while penetrating in a lossy medium. Following both from the new literature findings listed above, and from some exploratory work that tried to benefit from the leaky-wave antennas’ properties in order to create microwave applicators, such as [7], here, to achieve our objectives, we designed a particular leaky-wave antenna (LWA), which we derived from the Menzel antenna [8,9,10], and it was demonstrated as a suitable antenna design for deeper penetration, given the large value of the amplitude of the attenuation vector achievable by this structure. Such an antenna design has been modified here to make it shorter so that it would be suitable for biomedical applications, and more specifically hyperthermia, at a frequency of 2.4 GHz.

## 2. Materials and Methods

The antenna has been designed and simulated by using the CST Microwave Studio Software licensed to the DIET Department of “La Sapienza” University of Rome, and all figures shown in the paper have been obtained either by producing them with such a software, or re-designing them, eventually using Matlab, with the addition of custom information, aimed at providing additional details which were not available in the original figures.

The antenna operating frequency is 2.4 GHz. This is because the 2.4 GHz band belongs to the Industrial Scientific and Medical (ISM) frequencies [11], and it makes it particularly easy to penetrate inside biological tissues, since the wavelength is much larger than the average electrical thickness (with respect to the dielectric constant) of skin or muscles.

### 2.1. Leaky-Wave Antenna Design

**Figure 1:**
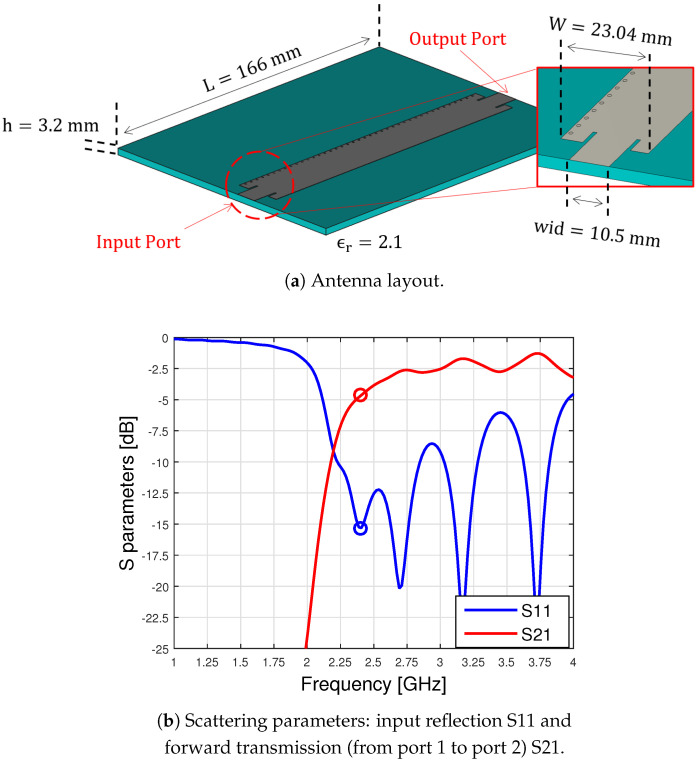
LWA design (**a**) and free space performances (**b**) in air (a vacuum).

Leaky-wave antennas (LWAs) are structures in which a propagating travelling wave loses power as long as it propagates. This happens due to asymmetries in general, periodically placed along the LWA, that disturb the normal flow of the energy producing radiation out of a guided mode [12,13,14]. The LWA design proposed here is based on the Menzel antenna, already considered in [2], and opportunely modified for the hyperthermia application requirements. The antenna in [2] has been designed to operate at 12 GHz. In that case, the chosen medium’s electric permittivity and magnetic permeability were chosen to be equal to the ones of a vacuum, but a finite non-zero conductance was added. That was a reasonable choice, since the main interest in those papers was purely theoretical, without addressing any specific application. As a result, we had to re-design the antenna for operating at 2.4 GHz. An LWA is usually several wavelengths long; therefore, keeping the form factor of the original design to operate at 2.4 GHz is not feasible (i.e., simply scaling the dimensions of a factor equal to 12/2.4). That is why we designed a particular antenna configuration, splitting the LWA into three sub-antennas. The single-element dimensions and performances in free space can be seen in Figure 1.

The antenna shown in Figure 1 operates at 2.4 GHz but is only 150 mm long, i.e., only a little longer than a wavelength. A proper transition of about 8 mm for each port is needed to match the antenna to 50Ω, leading to a total length equal to 166 mm.

Note that a longer antenna would not have been practical for hyperthermia applications. This design, which results in reduced efficiency, since only a fraction of the power supplied to the antenna is effectively radiated, will be modified in the following. It is understood that the remaining part is absorbed by the waveguide port placed at the end of the Menzel, so as not to let it radiate.

**Figure 2:**
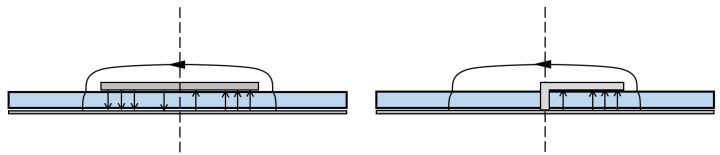
Electric field of the radiating mode on the input port of the Menzel antennas in Menzel antenna (**left**) and halved Menzel antenna (**right**).

With respect to the antenna presented in [2], this structure has been halved, exploiting the symmetry of the radiating mode (i.e., the first odd mode) through several via holes all along the symmetry axis. This simpler structure allows it to be easily fed by a 50Ω microstrip line. Moreover, halving the Menzel antenna allows us to suppress the dominant mode, as illustrated in [15]. The difference between the radiating mode excited in the classical configuration with respect to the halved Menzel antenna is visible in Figure 2.

Figure 2 represents an example of the image theory [16]. The S-parameters shown in Figure 1 represent the relationship between the power at the two ports. The first one is where the power is excited (one side of the antenna), and the second one is where the remaining part is absorbed (the opposite side of the antenna); see Figure 1. It is worth noting that, in this case, due to the losses of the net (i.e., the radiation of the antenna), the S-matrix is no longer Hermitian; this means that the sum squared of the $S_{11}$ and $S_{21}$ is not equal to 1 [17]. Of course, power that is not reflected nor transmitted is radiated.

**Figure 3:**
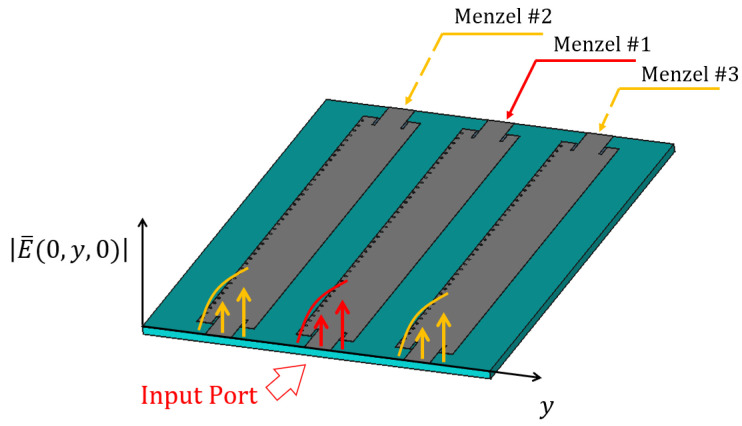
Considered leaky-wave antenna composed of 3 “sub-Menzel” antennas with field representation at the input ports. The picture illustrates qualitatively the field at the port for the three sub-antennas: the field for the central component, which is attached to the input port, is shown in red, for the other sub-antennas, the color yellow is used.

Since the $S_{21}$ in Figure 1 shows that the second port receives −4.9 dB of the power injected, it means that due to the shortness of the antenna, a consistent part of energy is still present in the dielectric. In order to obtain an efficient applicator, avoiding wasting such a consistent portion of provided power, and to extend the area under treatment, we modified the basic design, shown in Figure 1, by putting a power divider (in the figure below, a Wilkinson divider [17]) at the very end of the Menzel. To better understand this modification, let us consider the three identical Menzel antennas shown in Figure 3.

**Figure 4:**
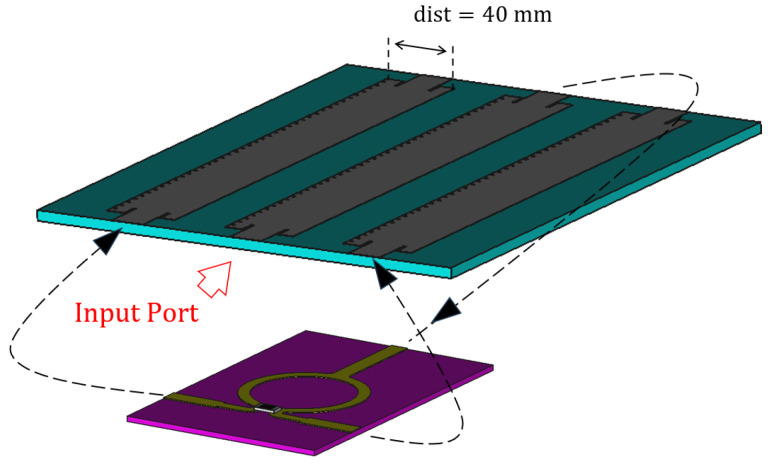
Leaky-wave antenna composed of 3 sub-Menzel antennas and a Wilkinson power divider. The dashed arrows indicate the direction of the electric field from and to the Wilkinson divider, while in red we indicate the position of the input port to the antenna.

Let us assume that the power generated by an RF source is connected to the input port of the “Menzel #1”, shown in Figure 3. Part of this power would be radiated along the “Menzel #1”, according to the S-Parameters shown in Figure 1. Instead of absorbing the remaining part, we have split it through a 3 dB power divider and used it to feed the input port of the sections of the antenna labelled as “Menzel #2” and “Menzel #3”, in Figure 4.

Concerning the choice of the 3 dB divider, it is important to observe that this choice was made to preserve the phase relation between the three Menzel components; in order to radiate properly, is important that all those are excited coherently. A simpler split of the two branches, as is customary in antenna arrays, could also be performed, but the Wilkinson divider assures the isolation between the two channels [18].

**Figure 5:**
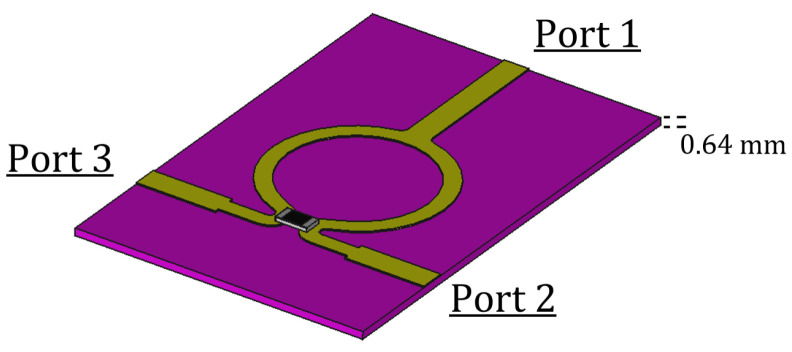
Wilkinson power divider design. Considered dielectric is RO5880 (Teflon).

**Figure 6:**
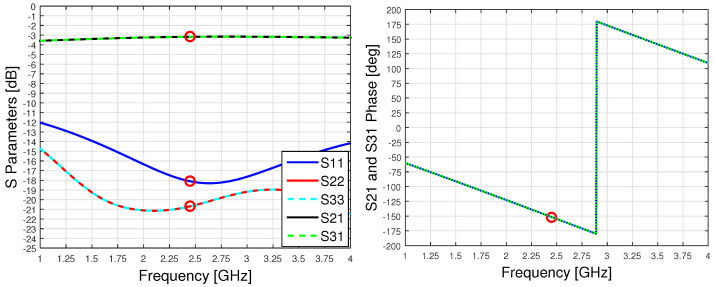
Wilkinson power divider: S-parameters of relevance (**left**) and phase relation (**right**). Circle markers are placed at 2.45 GHz.

The considered Wilkinson power divider is shown in Figure 5, while its performances are summarized in Figure 6. The S-parameters are normalized to 50Ω.

**Figure 7:**
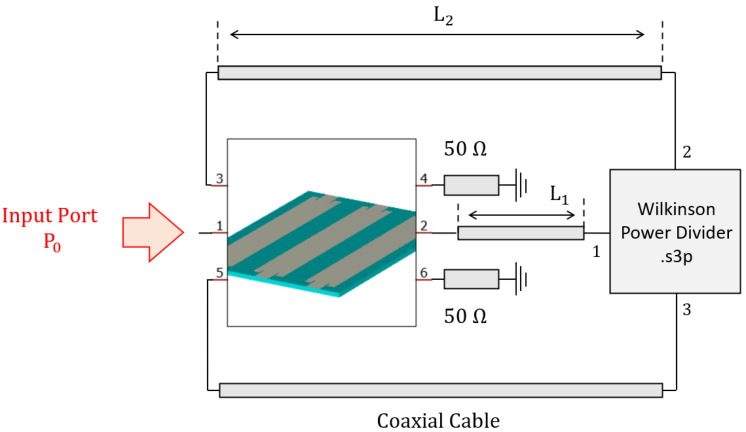
Circuital block scheme of the leaky-wave antenna structure.

Of course, in a possible antenna manufactured for this prototype, proper transitions and connectors should be considered. Under the schematic point of view, the structure is summarized in Figure 7.

Given the power of the input signal equal to $P_{0}e^{j\Delta \phi_{0}}$, the returned signal to port 5 and 5 of the LWA is

(4) $$P_{3}=P_{0}S_{21}^{LWA}S_{21}^{Wilkinson}e^{j\left[\Delta \phi_{0}+\arg\left(S_{21}^{LWA}\right)+arg\left(S_{21}^{Wilkinson}\right)+\Delta \phi^{cable_{2}}\right]},$$

where the cables losses have been neglected. In order to ensure the good behavior of the structure, it is important that the returned signal is coherent with the input signal. This happens if

(5) $$\Delta \phi_{0}+arg\left(S_{21}^{LWA}\right)+\Delta \phi^{cable_{1}}+\arg\left(S_{21}^{Wilkinson}\right)+\Delta \phi^{cable_{2}}=\Delta \phi_{0}$$

Or, equivalently,

(6) $$\Delta \phi^{cable_{1}}+\Delta \phi^{cable_{2}}=\frac{2\pi }{\lambda }\left(L_{1}+L_{2}\right)=-arg\left(S_{21}^{LWA}\right)-\arg\left(S_{21}^{Wilkinson}\right)$$

The latter represents the design law for the cables’ length.

**Figure 8:**
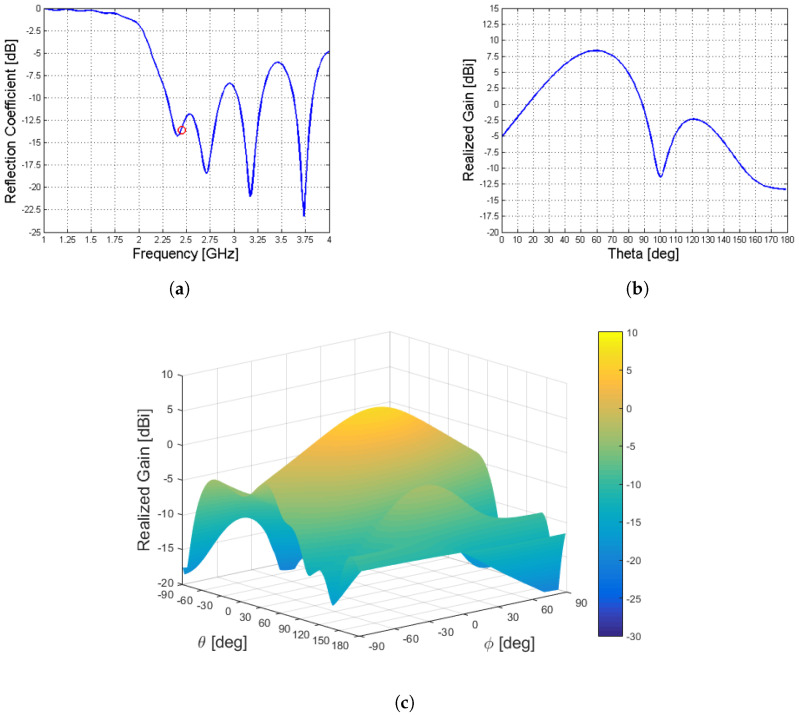
Reflection coefficient (**a**) and realized gain, (**b**,**c**), of the considered structure. Different colors, in (**c**), represent different amplitudes for the realised gain in dB, according to the scale on the right-hand side of the picture.

In Figure 8 are shown the reflection coefficient and the realized gain of the structure presented in Figure 7.

The realized gain $G_{r}$ for the loss-free antenna designed has been obtained by simulating the antenna in CST in free space (in a vacuum) and in the absence of the simulated lossy tissues. This simulation has been carried out to evaluate the antenna characteristics and to validate its design against that of the original Menzel antenna.

(7) $$G_{r}=4\pi \frac{\mathrm{power}\;\mathrm{radiated}\;\mathrm{per}\;\mathrm{unit}\;\mathrm{solid}\;\mathrm{angle}}{\mathrm{total}\;\mathrm{radiated}\;\mathrm{power}}\left(1-{\left|\Gamma \right|}^{2}\right),$$

where Γ is the impedance mismatch loss [19].

Eventually, a Monte Carlo analysis [20] was carried out to evaluate the impact of the cables and manufacturing tolerances over the antenna radiation mechanism. The analysis has been realized considering 1000 iterations and a uniform distribution for the tolerances of the phase and the amplitude mismatch on the returned signal, with respect to Figure 7. The idea is to consider a cumulative error vector that takes into account the non-ideality and asymmetries of the cables and the Wilkinson branches. For the analysis, a phase mismatch of $\left[-5^{\circ };+5^{\circ }\right]$ and a power mismatch of $\left[-2;+2\right]$ dB were considered between Menzel antennas #2 and #3. It is worth noting that the considered intervals are wide for modern technological capabilities.

**Figure 9:**
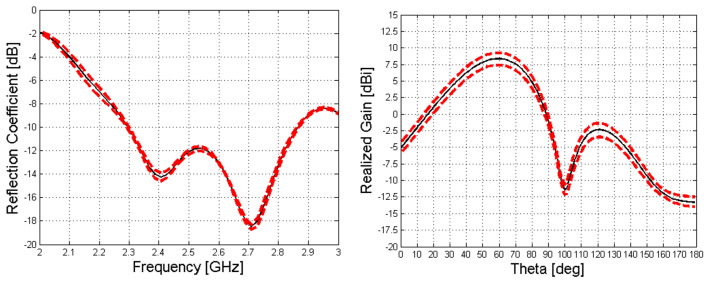
Reflection coefficient (**left**) and realized gain (**right**) of the 3 Menzel LWAs. The black curve represents the nominal value, and the red dotted ones represent the maximum and minimum of the Monte Carlo analysis.

In Figure 9 can be seen the interval of confidence in which the antenna’s performances are guaranteed with the considered intervals of phase and amplitude matching.

**Figure 10:**
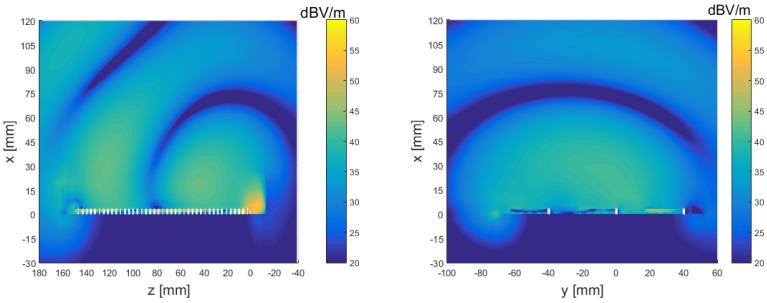
Near (electric) field generated by the considered leaky-wave antenna.

While we aim to design an antenna for near-field application, the far-field diagram still provides some insight into the quality of the obtained antenna design and provides the LWA pointing angle. In fact, to exploit the benefits of the deep-penetration effect, precise values of α and β are required. Calculating them directly in the near field may be very difficult. But for an LWA, these two values are bound to the far-field performances, i.e., the pointing angle and the beamwidth [12]. In Figure 10, we show the electric field amplitude in the proximity of the antenna (near field) which is relevant for the targeted hyperthermia application.

### 2.2. The Biological Tissues

To investigate the chance of using this novel structure in hyperthermia applications, we referred to the biological tissue models presented in [21,22,23,24]. Their electric permittivity vs. frequency is shown in the figures below, following the Cole–Cole model [24]. The considered stratification is as follows:

- 1.6 mm of skin;
- 7 mm of fat;
- 35 mm of muscles.

**Figure 11:**
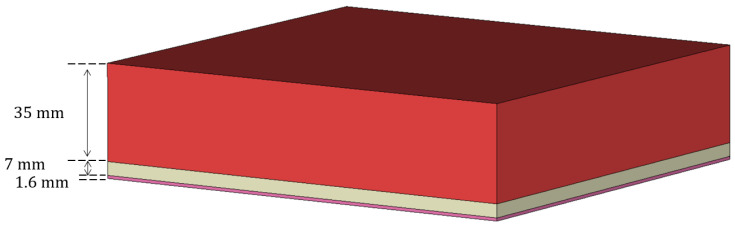
Considered tissue stratification.

Figure 11 represents the geometry for the biological tissues considered, while Table 1 and Table 2 illustrate their electromagnetic and thermal characteristics, respectively.

### 2.3. Assessment of the Heated Region

The simulations were carried out through a unidirectional solver, part of the suite CST-MW [25], that combines a FIT in the time domain [26,27] with a thermal transient solver that elaborates the power losses due to the electromagnetic fields. The thermal properties of the skin, fat, and muscles are chosen according to [24] and indicated in Table 2.

**Figure 12:**
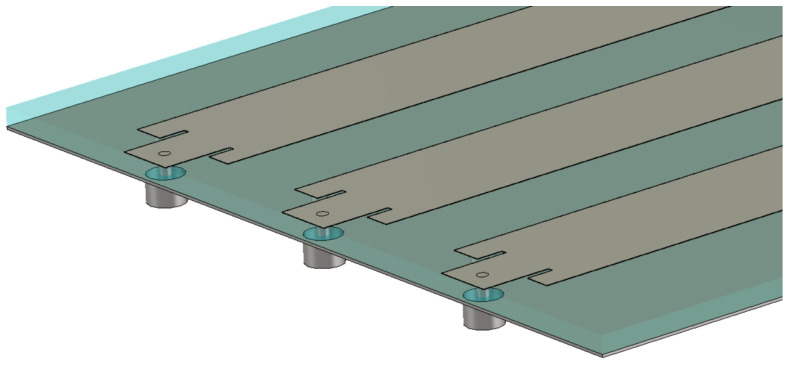
The 50Ω coaxial feeding network used to feed the LWA from below. The dielectric is represented as transparent.

The biological stack-up has to be placed in the near field of the LWA to experience an improved penetration due to the improper wave. The use of waveguide ports as feeding structures in this setup results in challenges, since they would electrically face a non-homogeneous medium that would affect the calculation or, in the worst case, they could interfere with the skin and the fat placed just above the antenna. That is why a different, and more realistic, feeding network had to be designed, i.e., a 50Ω coaxial feed inserted to excite the structure, as visible in Figure 12. Of course, the radiation mechanism remained unaltered.

**Figure 13:**
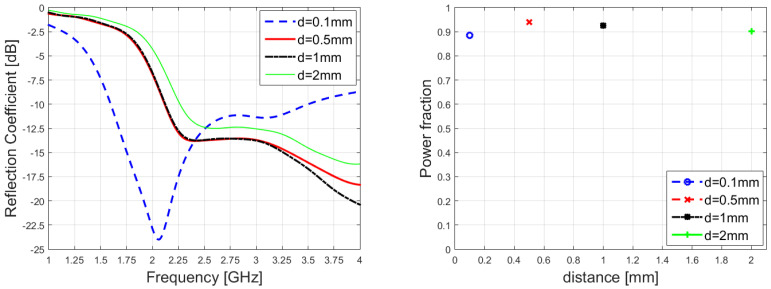
Reflection coefficient (**left**) and power dissipated inside the biological medium divided by the total power excited (**right**) of the leaky-wave antenna when placed in front of the tissue stratification.

The distance between the antenna surface and the biological medium has been optimized; different behaviors of the reflection coefficient have been assessed simply by varying the air gap. On the other hand, the reflection coefficient is not sufficient to guarantee that the power is correctly delivered. In fact, power that is not reflected to the coaxial connector could have generated a back-lobe or could have been coupled to other connectors. That is why another parameter has been taken into account to ensure that the distance would have really been optimized with respect to the biological stack up: the integral of the power density inside the skin, fat, and muscle. In Figure 13 is reported the result of the optimization. The value d = 0.5 mm has been chosen, due to the fact that the antenna proves to be very well-matched (left picture) and the power is correctly delivered inside the medium. The right side of Figure 13, in fact, represents the ratio between the power dissipated in the biological stack-up with respect to the stimulated one: more than 0.9. Of course, the remaining part of the power is absorbed by ports or by the radiative boundary conditions.

**Figure 14:**
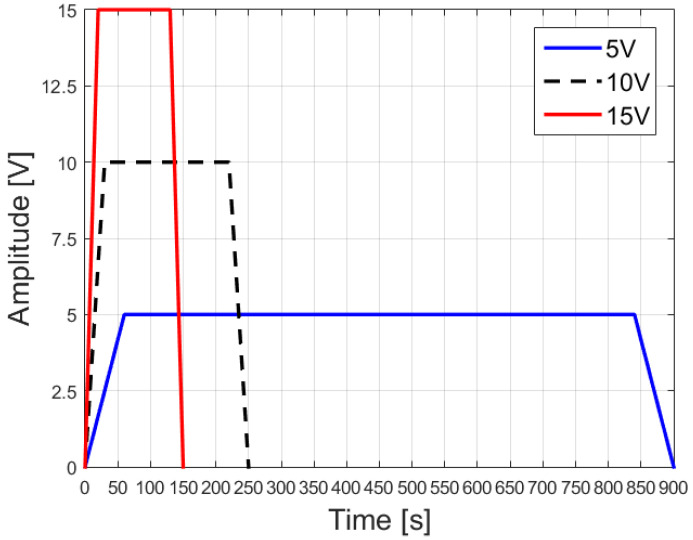
Excitation signals used for the simulation.

For the excitation signal, three different trapezoidal feedings were considered; they are shown in Figure 14.

The length is chosen to allow the temperature achieved by the skin tissue to reach 323 K. Once the desired temperature is reached on the surface part, the temperature distribution inside the medium is evaluated. The body temperature at rest has been set to 310 K in the simulation environment. Furthermore, to simulate the presence of blood flow, the blood perfusion coefficient has been activated. This simply enables the heat exchange mechanism between the tissue and blood, set to a temperature of 310 K.

## 3. Results

To better understand the simulation performed, we will give a little insight into the simulator tasks.

We firstly designed the electromagnetic model on the CST software, inserting all the electromagnetic and thermal characteristics of the media involved.

Then, we considered a power loss monitor at the frequency of interest, analyzing the problem with a time domain solver. The power loss monitor considered all the power radiated by the antenna and dissipated inside the tissue. After this step, the simulator used the obtained results as a source in the thermal solver, both the steady-state and the transient.

**Figure 15:**
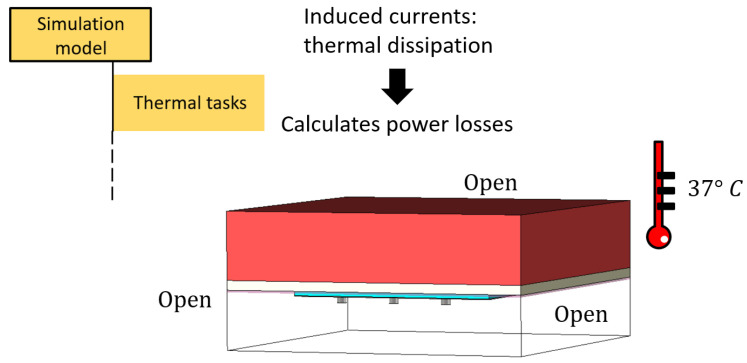
Schematic of the simulation task used in CST.

The simulation schematic is shown in Figure 15.

**Figure 16:**
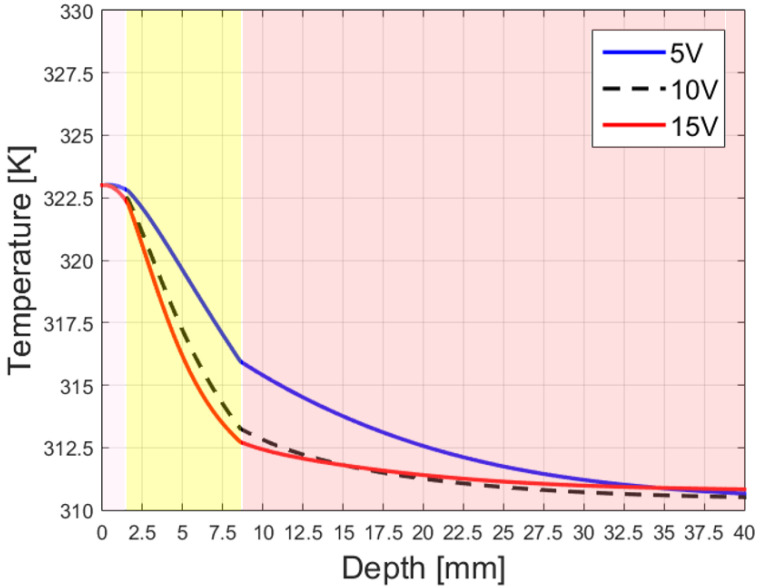
Temperature distribution, expressed in ${}^{\circ }K$, sampled along the stratification direction.

In Figure 16, the maximum value of the temperature reached by the body tissue vs. the depth inside it is illustrated. The different colors, stacked vertically at different relative depths, represent the different biological media (skin, fat, and muscle, respectively). In essence, the figure represents the behavior of the temperature along the stratification direction given different excitation signals. The behavior between the curves is similar; the temperature behavior is stable in the skin, remaining quite constant. Then, it drops in the fat quite linearly, eventually decaying exponentially inside the muscle. The optimization of the angle at the interface with the skin allows us to have an attenuation vector almost tangent to the separation surface between the skin and the air; this attenuation component needs to be preserved at the following interface (the one between skin and fat) for the well-known conservation of the attenuation component of electromagnetic fields at the interface between two media. Thus, the wave in the second medium cannot attenuate exponentially in the direction of propagation, increasing penetration. This “deeper penetration” is found to diminish as other interfaces are encountered, and so the attenuation decays almost exponentially in the direction of propagation in the third medium.

**Figure 17:**
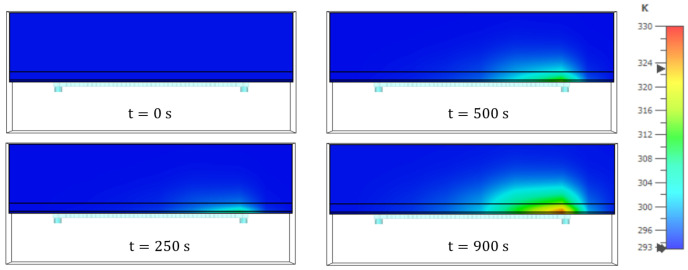
Temperature distribution, expressed in ${}^{\circ }K$, inside the biological sample for a 5V signal and at different timestamps.

Finally, in Figure 17 the temperature distribution expressed in ${}^{\circ }K$, inside the biological sample is also illustrated for different timestamps, to provide an insight into the change of temperature in time caused by the designed antenna in the sample tissue theoretical model.

## 4. Discussion

While the design of leaky-wave antennas for hyperthermia applications has been presented in the literature, even recently (see for instance [28,29]), the novelty of this article consists of proposing a structure that benefits from previous studies on deep penetration, allowing us to generate an electromagnetic wave that has an attenuation vector parallel to the separation surface between air and skin ($\zeta_{2}=90^{\circ }$), thus allowing a constant temperature in the skin, as shown in Figure 16. This result is significant, as it demonstrates that it is possible to shape the attenuation of the transmitted wave in practical antennas, overcoming some of the challenges evident in the previous literature on deep penetration, and in particular, the longitudinal dimension of the Menzel LWA necessary to achieve such an angle, which proved to be too large at the frequencies employed here. The antenna presented here aims to optimize the penetration in the skin and to minimize the probability of burns, by shaping the attenuation angle between air and skin. In doing so, we modify the attenuation component of the transmitted vector, obtaining a tangential component that must be preserved and increasing the penetration with respect to the case of normal incidence with a homogeneous wave. Anyway, the overall penetration could be further optimized, because the choice made did not optimize the transmitted wave at the interface between skin and fat or the one between fat and muscle. An alternative objective could be to reach the optimum overall penetration, rather than optimizing the temperature at the skin. In this case, the optimum incident angle at the first interface may not be a deep-penetration angle for any particular interface, but it could result in the best compromise. Clearly, doing so, a higher temperature on the skin surface would be expected.

Past studies have shown very good agreement between numerical models of tissues and real tissues [22]. Other studies have also demonstrated excellent agreement between the prediction obtained from numerical models and experimental verifications of applicators based on leaky-wave antennas; see [30]. However, while the models employed here represent a good average for typical values in the body, it is known that electromagnetic parameters can vary for factors such as the water content of the local tissue, or the skin thickness [23]. Therefore, a possible future antenna prototype may need specific optimizations for the region of interest. This optimization may also simply consist in modifying slightly the frequency to benefit from the frequency-scan properties of LWAs, to optimize the radiation angle for the region chosen. The antenna prototype shall also need to take into account constructive factors such as the tolerances for the materials employed. Finally, a prototype may need to take into account aspects typical of the hyperthermia treatment that are not within the scope of this paper, such as the integration of the antenna with other medical equipment, or the shape that optimizes the patient’s comfort and mobility.

## 5. Conclusions

In this paper, we presented an LWA for hyperthermia, and we tried to benefit from the deep-penetration effect to reduce the temperature at the surface of the skin, improving the heat distribution. The antenna designed shows that temperature in the skin is able to remain constant, and then start decreasing when the muscle is found, showing overall encouraging results. Some challenges have also been faced and resolved in terms of typical LWA lengths and efficiency that would affect the ideas presented in previous papers. Also, some of the manufacturing problems that may arise in such a design have been analyzed and commented on. Anyway, some future studies will be necessary for finding a good compromise between attenuation at the first interface (air–skin) versus the penetration at the second interface (skin–muscle) to allow an overall better result. Furthermore, the tissue composition that has been considered is sufficiently generic for this analysis. In future work, a particular body zone may be analyzed and optimized with respect to the typical thickness of the biological tissues involved. Finally, those results will have to be validated by performing prototype manufacturing and testing.

## Figures and Tables

**Table 1:** Electromagnetic properties for the selected biological tissues.

Tissue	$\varepsilon_{r}$	$\sigma \left[S/m\right]$
Skin	38.1	1.44
Fat	5.29	0.10
Muscle	52.8	1.171

**Table 2:** Thermal properties for the selected biological tissues.

Tissue	Thermal Diffusivity $m^{2}/s$	ρ $\left[{\mathrm{kg}/m}^{3}\right]$	Heat Capacity [KJ/(Kg·K)]	Thermal Conductivity [W/(m·K)]
Skin	$9.8e^{-8}$	1109	3.391	0.37
Fat	$9.8e^{-8}$	911	2.348	0.21
Muscle	$1.31e^{-7}$	1090	3.421	0.49

## Data Availability

Not applicable.

## Abbreviations

The following abbreviations are used in this manuscript:

LWA	Leaky-Wave Antennas
FIT	Finite Integration Technique
